# Two-photon excitation fluorescence in ophthalmology: safety and improved imaging for functional diagnostics

**DOI:** 10.3389/fmed.2023.1293640

**Published:** 2024-01-03

**Authors:** Vineeta Kaushik, Michał Dąbrowski, Luca Gessa, Nelam Kumar, Humberto Fernandes

**Affiliations:** ^1^Institute of Physical Chemistry, Polish Academy of Sciences, Warsaw, Poland; ^2^International Centre for Translational Eye Research, Institute of Physical Chemistry, Polish Academy of Sciences, Warsaw, Poland

**Keywords:** two-photon excitation fluorescence, diagnostics, ophthalmology, functional imaging, non-invasive, adaptive optics

## Abstract

Two-photon excitation fluorescence (TPEF) is emerging as a powerful imaging technique with superior penetration power in scattering media, allowing for functional imaging of biological tissues at a subcellular level. TPEF is commonly used in cancer diagnostics, as it enables the direct observation of metabolism within living cells. The technique is now widely used in various medical fields, including ophthalmology. The eye is a complex and delicate organ with multiple layers of different cell types and tissues. Although this structure is ideal for visual perception, it generates aberrations in TPEF eye imaging. However, adaptive optics can now compensate for these aberrations, allowing for improved imaging of the eyes of animal models for human diseases. The eye is naturally built to filter out harmful wavelengths, but these wavelengths can be mimicked and thereby utilized in diagnostics via two-photon (2Ph) excitation. Recent advances in laser-source manufacturing have made it possible to minimize the exposure of *in vivo* measurements within safety, while achieving sufficient signals to detect for functional images, making TPEF a viable option for human application. This review explores recent advances in wavefront-distortion correction in animal models and the safety of use of TPEF on human subjects, both of which make TPEF a potentially powerful tool for ophthalmological diagnostics.

## 1 Introduction

Diagnostics, and early diagnostics in particular, are crucial in ophthalmology. Many eye diseases are progressive and, after a certain point, several are irreversible. Thus, early detection of symptoms can significantly increase the chances of proper treatment, subsequently halting, or at least considerably slowing, the progression of the disease. Basic methods of diagnostics rely on direct visualization and subjective questioning of the patient. Such methods have been complemented with structural imaging, such as optical coherence tomography (OCT) (1, 2), in which technology has advanced to allow for imaging of morphological changes, distinguishing healthy from sick/damaged eye components. However, there is a need for non-invasive functional imaging that will enable several advanced diagnostic approaches, including measurement of structural changes upon stimulus, for example with optoretinography (ORG) (3–5); direct assessment of biochemical changes, with two-photon (2Ph) imaging (6); and detection of human retinal fluorophores *in situ* in the interphase between the retina and the retinal pigment epithelium (RPE) layer to recognize imbalances in the visual cycle (6). 2Ph imaging is also helpful in examining the collagen arrangements in the human cornea and sclera and the non-collagen limbus ultrastructure of the trabecular meshwork (7).

Advances in 2Ph imaging have alleviated safety concerns and allowed for its potential use in *in vivo* diagnosis of corneal and retinal diseases, at a minimum. Here we briefly introduce the two-photon excitation fluorescence (TPEF) technique and its use in ophthalmology, highlighting some of its most promising applications.

## 2 2Ph imaging

**Figure 1:**
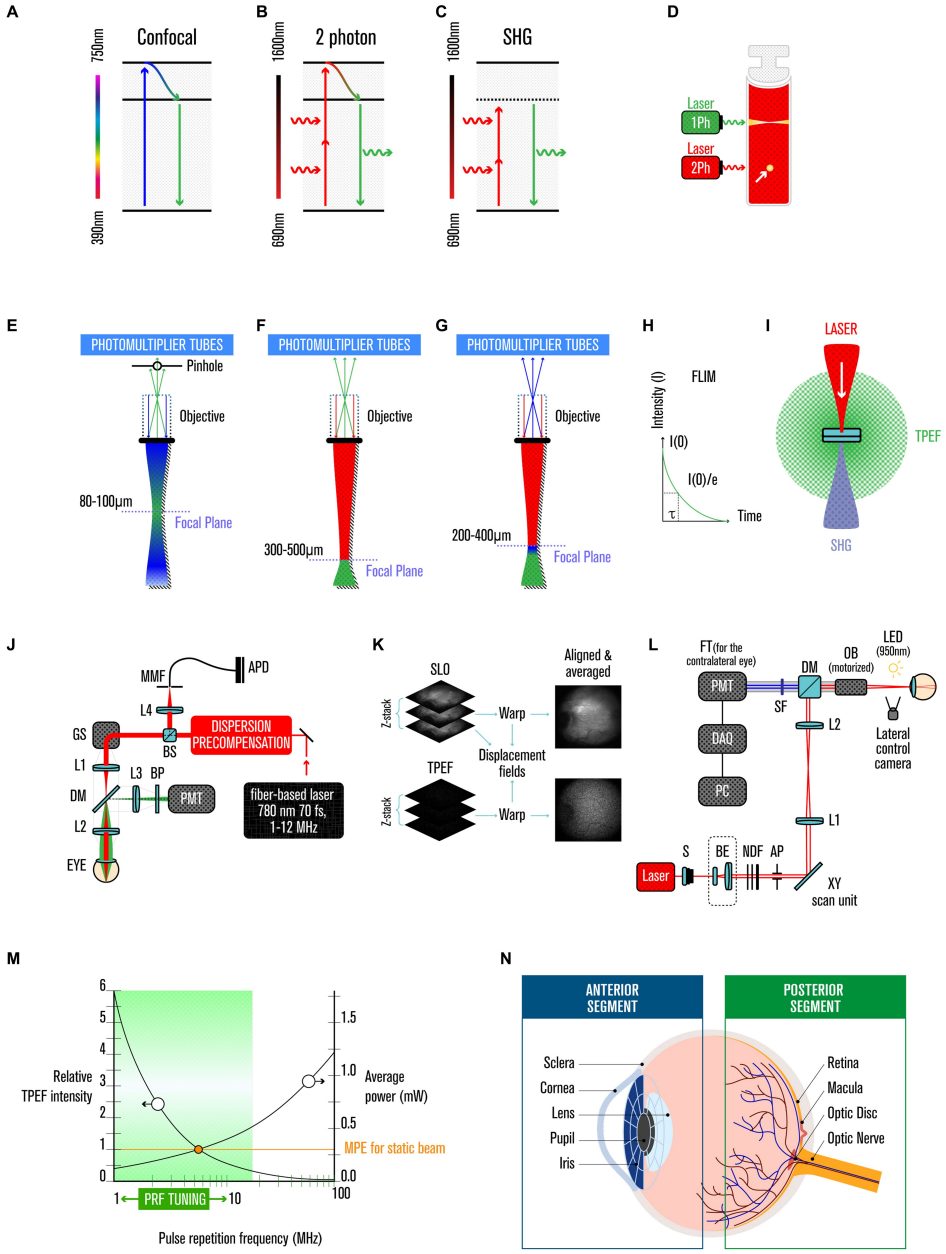
Two-photon imaging. **(A)** Jablonski diagram of fluorophore excitation by single, **(B)** two-photon, and **(C)** secondary-harmonic generation (SHG). **(D)** comparison of one and two-photon (*yellow arrow*) excitation profiles. **(E)** focal plane range by single, **(F)** two-photon, and **(G)** SHG. **(H)** FLIM histogram of photon counts versus arrival time after the laser pulse. **(I)** two-photon excited fluorescence and SHG are isotopically emitted. **(J)** experimental TPEF-SLO setup, and its **(K)** imaging processing. **(L)** experimental SHG setup. **(M)** relative TPEF intensity as a function of pulse repetition frequency. **(N)** anatomy of the human eye.

2Ph imaging is a fluorescence image technique based on the fact that two photons interact with molecules quasi-simultaneously (on the order of femtoseconds), in an incoherent fashion, with the combined energy matching the energy gap between the ground and excited states, and the absorption occurring most strongly near the focal plane, where the photon flux is highest (8) (Figures 1A–D).

This is a very rare event, but can be largely boosted by the use of ultrafast pulsed laser excitation in the range of 10^20^–10^30^ photons/(cm^2^⋅s) (7), with the number of photons absorbed per fluorophore per pulse (*n_a_*) is given by:


$$n_{a}\approx \frac{P_{0}^{2}\delta }{\tau_{p}f_{p}^{2}}{\left(\frac{{\left(NA\right)}^{2}}{2\hbar c\lambda }\right)}^{2}$$

with *τ_p_* being the pulse duration, *δ* is the fluorophore’s two-photon absorption cross section (~10^−58^ m^3^ per photon) at wavelength *λ*, *P_0_* being the average laser intensity, f *_p_* being the laser’s repetition rate, *NA* being the numerical aperture of the focusing objective, and *ℏ* and *c* being the Planck’s constant and the speed of light, respectively (8, 9). With such small cross-section and with a pulse duration of 100 fs and a lens numerical aperture ≈ 1.4, Denk and coworkers calculate that an average incident laser power of ~50 mW would saturate the fluorescence output at the limit of one absorbed photon pair per pulse per fluorophore (9).

Currently the 2Ph absorption, after non-radiant energy relaxation, is explored in medical context in three main ways: (i) in the emission of a single photon in the visible range via fluorescence emission (TPEF), (ii) or in case of instantaneous process and no energy lost, in a second-harmonic generation (SHG) scattered UV light (7, 10), and (iii) in fluorescence lifetime imaging microscopy (FLIM) (Figures 1E-I).

TPEF has several unique advantages over conventional fluorescence microscopy, such as reduced phototoxicity, increased penetration depth and improved spatial resolution. The first application of TPEF to biological samples occurred in 1990 (9); since then, it has been used for various medical applications, including ophthalmology (11) (Figures 1J–K).

SHG is a coherent process, thus the phase and polarization of the generated photon are related to those of the incident photons, bringing advantages over other imaging techniques such as high contrast, minimal photobleaching, deep penetration depth, and allowing label-free imaging. SHG was first demonstrated in 1961 (12) and can be used for high-resolution optical microscopy in biology and the medical sciences (Figure 1L) (see Supplementary Material).

FLIM extracts the lifetime from the fluorescence emission (10), allowing the separation of fluorophores that just by the intensity at a particular wavelength would not be distinguishable (10, 13–17) (see Supplementary Material).

Such unique characteristics grant TPEF an amazing potential for use in both structural and functional imaging. In ophthalmology, progress has been made in evaluating the applicability of 2Ph in the clinic for corneal and retinal analysis. Moreover, TPEF, as a non-invasive and label-free optical imaging technique, allows the characterization of specimens without interference from the biochemical composition and/or physiological state of the samples (18).

## 3 Safety

Safety has been a critical element in developing 2Ph imaging for application in ophthalmology, especially when imaging the retina where melanin-mediated interference can preclude detection of the signal of interest. Thus, advances in manufacturing the laser source (with spatial, temporal and spectral properties finely modulated) were key in maximizing the signal and minimizing the exposure to laser light (6, 19); i.e., very intense but short pulses (which, if continued, would vaporize the biological samples), at a high repetition rate, that produce high instantaneous energy but low average (20). The probability of TPEF events increases with photon flux, thus requiring powerful lasers (or less powerful if the laser produces short fs pulses, making the instantaneous intensity very high). Despite the use of these high-intensity short pulses, eye tissue interrogations remain within safe limits because of the minimal cross-section of material illuminated, and the very limited time period over which the pulses are delivered (Figure 1M). Technology tested in mice revealed the possibility of extracting biochemical information using TPEF imaging, within safe limits and without any perceived damage judged by several criteria, including *in vivo* imaging using SLO and OCT; retinal function assay by ERG; and *ex vivo* quantification of rhodopsin and 11-cis retinal by immune histology and TPEF imaging (6, 21). To ensure a more significant safety margin for use in human retinas, Palczewska et al. (21) further diminished the potential sources of photodamage by decreasing the pulse repetition frequency (PRF) (17), and by adding other design features in the setup (22, 23). In more detail, Palczewska and colleagues’ setting is now capable of recording retinoids derivative in human retinas using average light power of only 0.3 mW for an exposure time of 40 s per measurement (that is sufficient to collect 30 frames), when considering a NA of 0.22, is well below the safety limits prescribed by the American National Standard for Safe Use of Lasers, ANSI Z136.1–2014. Using a laser of λ = 780 nm, a pulse duration of 76 fs, and imaging a squared retinal area of 17.6° and using the equation:


$$RE=\frac{P\;.\;T_{\text{exp}}}{A_{\mathrm{retina}}}$$

For the retinal exposures (RE), where *P* is average excitation power, *T_exp_* is exposure time and *A_retina_* is the area of the exposed retina (see Supplementary Material).

In another study, Avila et al. (7) used a high-repetition infrared laser and, keeping the maximum laser power (MP) within safety limits, obtained 2Ph images while protecting both the cornea and the retina (7). For their setting, and considering both the International Commission on Non-Ionizing Radiation Protection (ICNRP) (24), and the ANSI Z136.1–2000 (19), Avila and co-workers an MPE of 3.49 W/cm^2^ was determined, and they collect their images with exposure times of 0.42 s (7).

## 4 Image improvement

Adaptive optics (AO) is a technique that at its core uses a wavefront sensor to measure the distortion in an optical wavefront, and corrects them using a deformable mirror, thus in ophthalmology AO corrects for eye aberrations (25). Additionally, one impact of reducing the pulse duration in TPEF imaging is the increased phase distortion, caused not only by the tissues but also by the optical component due to the increase in the pulse spectral bandwidth, which needs to be compensated for (6, 26). Another impact to some extent is wavefront distortion, due to intermediate tissue layers; this problem may be overcome using AO (6, 27–29). Several innovative approaches to solve such problems include Differential Aberration Imaging (DAI), which computes out tissue-induced aberrations (30); real time Iterative Aberrations Correction, which involves iterative algorithms to adjust the optical elements and thus the quality of the images (31); and Offline Aberration Corrections that involve post-processing corrections to improve image quality (32). However, adaptive optics needs to correct the wavefront aberrations without inducing much photobleaching or increasing the exposure of the sample. This refinement was achieved for 2Ph microscopy in 2009 (33), and it was used for other imaging modalities in ophthalmology practice in 1997 (34) and in a clinical setting in 2000 (35). More information on adaptive optics, including its use on different cells and tissues, clinical applications, and current limitations and perspectives, can be found in a review by Akyol and colleagues (36).

## 5 TPEF use in ophthalmology

The eyes are a very sensitive organ that in parallel to detect a large spectrum of radiation and over a large range of intensities, needs to protect its delicate fabric of different tissues and cells from radiation damage. UV light is particularly detrimental (it can lead to irreversible changes in the transparency of the human cornea and lens (37)), but is filtered before it reaches the sensitive photoreceptors by the cornea and lens, and it is also quenched by the presence of melanin, that works not only as a photo-screen but also as an antioxidant (38).

In TPEF, two less-energetic (i.e., near IR range) photons are used to excite molecules instead of a single high-energy photon (i.e., in the UV range), thus bypassing the usage of UV illumination limitations. This approach provides multiple advantages for using TPEF as a diagnostic tool, including: (i) it causes less damage (8–10), as it has a quadratic dependence on the excitation intensity (instead of the linear dependency of one-photon excitation); (ii) it has a more profound penetration power in tissues because the excitation is limited to the focal volume with high photon density (8–10, 20) (Figures 1E–F), and there is decreased scattering (8–10, 32); and (iii) in ophthalmology, it avoids the sequestration of UV light by the anterior segment of the human eye (39, 40) (Figure 1N).

The eye is composed of anterior and posterior segments (Figure 1N). The anterior segment contains the cornea, iris, ciliary body, and lens; and the posterior segment comprises the retina, choroid and optic nerve. Between the cornea and the optical nerve, the eye is coated by a white fibrous tissue called the sclera, which is the supporting structure that shapes and protects the eyeball. The posterior segment is responsible for detecting a light stimulus and its converting it into an electrical stimulus that the brain can process to generate images. In contrast, the anterior segment focuses the light beam onto the retina and blocks damaging UV light.

The cornea is the outermost anterior part of the eye, and it is transparent and avascular to allow light transmission and refraction (10). Its thickness varies from 500 μm in the center to 700 μm toward the periphery. The cornea is comprised of five main layers (epithelium, Browman’s layer, stroma, Descemet’s membrane, and endothelium). The stroma accounts for ~90% of the corneal thickness, and is composed of stacked collagen lamellae (7, 10). SHG allows the visualization of non-centrosymmetric molecules, such as collagen (7, 41, 42). It was first performed *ex vivo* (41, 43–47) and later performed *in vivo* in rabbits and humans (7, 41, 48). *Ex vivo* work indicates that TPEF can distinguish between pathological and non-pathological cornea for diseases such as keratoconus (KC), Acanthamoeba keratitis (AK), and stromal corneal scars (SCS), based on collagen fiber organization and stromal autofluorescence (AF) lifetime, as well as cell morphology and metabolism (41). Moreover, TPEF can detect changes in the ratio of free and protein-bound NAD(P)H to infer a decrease in cell metabolism (20, 41); and other clinical applications have been investigated over the years, as reviewed in 2022 (10). Recently, human eyes were evaluated *in vivo*, and information on the collagen arrangements and morphological features was obtained for the cornea, sclera, and trabecular meshwork (7). The latter revealed small features that may play a role in intraocular pressure regulation and glaucoma diagnostics, and mirrored the observations of *ex vivo* studies (7, 49–51).

As indicated, the posterior part of the eye includes the retina, where light stimuli are converted into electric stimuli that are further processed by the brain to generate images. One key piece of the process is the visual cycle, which contains many natural fluorophores that can be detected (6). However, their short excitation wavelengths do not permit the use of traditional one-photon imaging, as UV light will cause eye damage (22). As the anterior segment filters the light, 2Ph delivered in femtosecond pulses can interrogate such molecules in the retina (6, 17, 52, 53). TPEF imaging can detect several disease-specific biomarkers (52, 54, 55), as well as light- and age-induced retinal defects since they cause changes in retinoid metabolism (6, 56). More specifically, this imaging technique was employed to identify lipid droplets with a high concentration of retinyl esters (57), which over-accumulate in Rpe65−/− mice and are absent in Lrat−/− mice (58, 59). The ablated genes in these mouse models code for two key enzymes in the visual cycle, RPE65 and LRAT, respectively. Rpe65−/− mice are a model for Leber congenital amaurosis, and Lrat−/− mice are a model for retinitis pigmentosa (6, 60). In animal models, TPEF imaging can distinguish aberrant bisretinoid patterns caused by Stargardt disease (61). In addition to the disease setting, during ageing some retinoids form bisretinoids that are fluorescent and thus can be analyzed with 2Ph ophthalmoscopy (62). From a therapeutic point of view, TPEF imaging is also potentially relevant, as it can help to track restoration of visual function. Examples include monitoring gene therapy of pathogenic mutations in the Rpe65 gene (63), tracking the drug-induced diminution of bisretinoids in a mouse model of Stargardt disease (61), and probing the protective effects of pharmacological agents on photoreceptor-induced light damage (64).

## 6 Other medical applications and comparison to other ocular techniques

TPEF is effective in exciting aromatic amino acids and other fluorophores present in the cells such as NAD and FAD allowing the analysis of living tissues (65, 66), thus getting used in different fields other than ophthalmology including neurobiology for studying neuronal signaling processes (67), oncology for investigating metabolism in cancer cells (68), and in the field of embryology for studies on living embryos (69). 2PEF has proved to be useful also in the field of immunology, thanks to the ability to give information about living cells revealing several mechanisms of the adaptive immune response (70).

Diagnostics of the cornea have historically relied on imaging techniques such as a slit-lamp microscope and specular and confocal microscopy, further supported by anterior segment OCT and corneal topography (41). These techniques can detect alterations of cell morphology, but they cannot monitor changes in the metabolism of cells or the structural organization of the stroma (41).

In terms of structural imaging of cornea, TPEF is superior to OCT and specular microscopy (or reflectance confocal microscopy) allowing the visualization of collagen fibers, and overall giving better contrast/resolution of the different corneal layers (7). Also, functional imaging is impossible with standard OCT.

Like the cornea, the retina has been examined with various imaging techniques, such as color photography, narrow-band reflectance photography, scanning-laser ophthalmoscopy (SLO) and fundus autofluorescence (6). Despite offering 2D-structural information, or 3D information when OCT is used, and allowing scrutiny of the retinal layers (6), these techniques do not offer insights into metabolic status or other functional information, and the information they provide does not correspond unfailingly to visual acuity (71). 2Ph imaging permits the measurement of the retinoids *in vivo* and thus facilitates an evaluation of the health of the visual cycle.

Fundus Autofluorescence (FAF) captures the natural fluorescence emitted by retinal pigments, thus providing insights into retinal health and metabolic changes, and applicable in conditions like macular degeneration and inherited retinal diseases (72). FAF and TPEF, applied to the posterior segment of the eye, share many similarities, but there are key differences. The more sticking is the fact that TPEF can pinpoint fluorophores active in the visual cycle, and not only passive ones such as melanin (73), and components of lipofuscins (22), and using lower Res, 1.76 J/cm^2^ for a multiple-day exposure (22) versus 15 J/cm^2^ (74), and 20.4 J/cm^2^ (75), using near-infra-red autofluorescence (NIR-FAF), thus also avoiding photochemical degradation of retinoids (76, 77).

## 7 Future perspectives

Two current limitations of TPEF are the slow acquisition times and low signal-to-noise ratio. Current developments with the use of multiple detectors offer the promise that faster and more efficient imaging collection (78). Using lasers that are frequency encoded (FE), Heuke and colleagues, offer now the possibility of excitation discrimination (in addition to the already available emission discrimination) and thus enable an unprecedented number of fluorophores to be imaged simultaneously (78).

There are a few areas where we think 2Ph imaging will have a significant impact in ophthalmology. Namely advances in the imaging of other retinal layers such as retinal ganglion cells with fiber-based two-photon fluorescence lifetime imaging ophthalmoscopy (FLIO) (79), or SHG to image the density and distribution of rhodopsin in rod photoreceptors (80). Moreover, 2Ph imaging can be used to assess corneas viability for transplantation (43), detect changes in the corneal shape and/or its mechanical properties and identify the initiation of loss of transparency, and thus future sight impaired or vision loss (7). Even when such a process occurs, since NIR excitation light penetrates cataractous lenses in a nondestructively way (22), 2Ph can nonetheless be used to access retinal function in elderly people, coincidentally more prone to suffer from illness on both eye segments. One final front where 2Ph will be used in the future is on drug impact and mechanism of action of drug action in retinal tissue (6).

## 8 Conclusion

In recent decades, and during recent years in particular, advances in TPEF technology, including adaptive optics and safety improvements, have raised its status and highlighted it as a potentially powerful tool with ophthalmological clinical applications, especially due to its functional imaging capacity. There are several challenges, however, to its widespread use, including the cost and the complexity of the current instrumentation, the need for self-assembly of the equipment, the costs and slow-speed of running adaptive optics, and the limited data from human *in vivo* tests (due to safety concerns that now seem to have been addressed). Nevertheless, enough of the limitations on the technology have been overcome, so that TPEF may now be considered a valuable potential tool for clinical diagnostics, to distinguish healthy from pathological eyes in a non-invasive and label-free manner (Table 1). TPEF can be used for structural and functional imaging of both the anterior and posterior segments of the eye, and it may be relevant for diagnosing and tracking treatment efficacy for ocular diseases including keratoconus, keratitis, bulbous keratopathy, fibrosis, Stargardt disease, and retinitis pigmentosa, among others.

**Table 1:** Advantages and disadvantages of TPEF in ophthalmology.

TPEF in ophthalmology	
Advantages	Disadvantages
Non-invasive	Price
Functional imaging	Safety concerns for animal/human usage
Decreased photodamage	Not available “off the shelf”
High penetration depth	Adaptive Optics, slow speed
High contrast	Image acquisition time
*in vivo*/*in situ*	Lower signal to noise (SNR) ratio
Dye-free	Complexity of interpretation

## Author contributions

VK: Writing – review & editing. MD: Writing – review & editing. LG: Writing – review & editing. NK: Writing – review & editing. HF: Writing – original draft, Writing – review & editing.
