# Corrigendum: Two-photon excitation fluorescence in ophthalmology: safety and improved imaging for functional diagnostics

**DOI:** 10.3389/fmed.2025.1572630

**Published:** 2025-02-19

**Authors:** Vineeta Kaushik, Michał Dąbrowski, Luca Gessa, Nelam Kumar, Humberto Fernandes

**Affiliations:** ^1^Institute of Physical Chemistry, Polish Academy of Sciences, Warsaw, Poland; ^2^International Centre for Translational Eye Research, Institute of Physical Chemistry, Polish Academy of Sciences, Warsaw, Poland

**Keywords:** two-photon excitation fluorescence, diagnostics, ophthalmology, functional imaging, non-invasive, adaptive optics

In the published article, there was an error in the **Funding** statement. It originally stated: [The author(s) declare financial support was received for the research, authorship, and/or publication of this article. The International Centre for Translational Eye Research (MAB/2019/12) project was carried out within the International Research Agendas programme of the Foundation for Polish Science co-financed by the European Union under the European Regional Development Fund. VK was the awardee of the fellowship within PASIFIC postdoctoral fellowship programme (Agreement No PAN.BFB.S.BDN.315.022.2022; Project No. DPE/2023/00007), co-funded by the European Union under Marie Skłodowska-Curie CO-FUND scheme (grant agreement No 847639), Horizon 2020.]. The correct Funding statement appears below.

