# Supplementary material for: Two-photon excitation fluorescence in ophthalmology: safety and improved imaging for functional diagnostics
Source: Front Med (Lausanne). 2024 Jan 3;10:1293640. doi: 10.3389/fmed.2023.1293640 (PMC10791900; doi:10.3389/fmed.2023.1293640)
Supplement: Supplementary file 1 [file Data_Sheet_1.PDF]

Supplementary Material

Two-photon excitation fluorescence (TPEF) in ophthalmology: safety and improved imaging for functional diagnostics

Vineeta Kaushik[1], Michał Dąbrowski[1,2], Luca Gessa[2], Nelam Kumar[2], Humberto Fernandes[2*]

[1]Institute of Physical Chemistry, Polish Academy of Sciences, Warsaw, Poland

[2]International Centre for Translational Eye Research, Institute of Physical Chemistry, Polish Academy of Sciences, Warsaw, Poland

*** Correspondence:**
Humberto Fernandes
hfernandes@ichf.edu.pl

Second Harmonic Generation (SHG) of endogenous proteins

Only non-centrosymmetric structures emit SHG light due to its non-zero second-harmonic coefficient, and its first application in biology was a 1986 study of collagen fiber orientation in rat tail tendons [1]. In proteins, at the molecular level, SHG originates from the hyperpolarizability of peptide bonds, where an electric field oscillating at a high frequency and reaching an harmonophore will lead to the induction of a molecular dipole by repeatedly pull the electrons back and forth:

$$P = P^{(0)} + \alpha E + \beta EE + \gamma EEE +$$

(Eq supp1)

where α is the polarizability of electrons of the peptide bond, E the incident electric field and β and γ the hyper- polarizabilities of the first and second order, respectively [2].

Fluorescence Lifetime Imaging Microscopy (FLIM)

During the average time that a fluorophore stays in the exited state, the intensity $I(t)$ decreases 1/e of its initial value. With the decaying intensity at time t be given by:

$$I(t) = \sum_i \alpha_i e^{-t/\tau_i}$$

(Eq supp2)

That is a first-order kinetics equation summed across all species, i, in the sample, and where α is the pre-exponential factor or the amplitude of the exponential function. For a multiexponential mixture of species the mean lifetime (τ_m) is the sum of each species lifetime (τ_i) weighted by fractional contribution of each species (α_i):

$$\tau_m = \sum_i \tau_i \alpha_i$$

(Eq supp3)

And, the number of excited molecules at a time t is given as:

$$n(t) = n(0)e^{-t/\tau}$$

(Eq supp4)

where $n(t)$ is the number of molecules in the excited state at time t [3].

TPEF safety

For the retinal exposures (RE), where P is average excitation power, T_{exp} is exposure time and A_{retina} is the area of the exposed retina, itself defined as:

$$A_{retina} \approx \frac{\pi}{4}(f_e \alpha)^2$$

(Eq supp5)

Where f_e is the focal length of the human eye (17 mm, Gullstrand's model eye) and α is the full angle of exposed area in radians (346 mrad in the work of Boguslawski and collaborators ([4]) gives a RE of 0.044 J/cm^2 (0.176 J/cm^2 for a day of measurements (=4 exposure of 40 second each) and 1.76 J/cm^2 for repeated exposures of the human eye (= to 160 seconds)).

Because fluorescence (F), in TPEF with pulsing light, is proportional to the square of average power (P_{avr}):

$$F \propto K \cdot \frac{g_p}{PRF \cdot \tau} \cdot P_{avr}^2$$

(Eq supp6)

where K is the constant dependent on concentration and type of fluorophore, wavelength and the collection efficiency of the system; g_p is a dimensionless constant dependent on the shape of the laser pulse; PRF is the pulse repetition rate; and τ is the pulse duration in the sample. Figure 1M helps to demonstrate that, because the average excitation power being limited in safety grounds (set by ANSI most restrictive limit standard) to a strictly limited maximum permissible exposure

(MPE) of 0.3 mW, and the pulse duration kept as short as possible by incorporating dispersion compensation, equation (Eq supp6) shows that only tuning PRF allows maximization of signal capture (ie. signal enhancement under the safety restrictions described above) with lower PRF generating more fluorescence photons at a given excitation average power. Lowering the PRF has the additional benefit of plasma formation and thermal effects related to the presence of melanin [5]. After TPEF exposures, human retinas show no damage as judged by blue-induced FAF (B-FAF), NIR-FAF, optical coherence tomography (OCT) and 1- and 2-photon perimetry [4], and more extensive safety studies done in animal models also did not detect any issues. The testes included in vivo imaging with SLO and OCT; measurements of rhodopsin and 11-cis-retinal; histology and 2P imaging of the RPE; retinal function assessment by ERG; and ex vivo microscopy imaging of retinal sections immuno-labeled for glial fibrillary acidic protein (GFA) [6].

Bibliography

[1] I. Freund, and M. Deutsch, Second-harmonic microscopy of biological tissue. Opt Lett 11 (1986) 94.

[2] A. Aghigh, S. Bancelin, M. Rivard, M. Pinsard, H. Ibrahim, and F. Legare, Second harmonic generation microscopy: a powerful tool for bio-imaging. Biophys Rev 15 (2023) 43-70.

[3] R. Datta, T.M. Heaster, J.T. Sharick, A.A. Gillette, and M.C. Skala, Fluorescence lifetime imaging microscopy: fundamentals and advances in instrumentation, analysis, and applications. J Biomed Opt 25 (2020) 1-43.

[4] J. Boguslawski, G. Palczewska, S. Tomczewski, J. Milkiewicz, P. Kasprzycki, D. Stachowiak, K. Komar, M.J. Marzejon, B.L. Sikorski, A. Hudzikowski, A. Gluszek, Z. Laszczych, K. Karnowski, G. Sobon, K. Palczewski, and M. Wojtkowski, In vivo imaging of the human eye using a 2-photon-excited fluorescence scanning laser ophthalmoscope. J Clin Invest 132 (2022).

[5] G. Palczewska, J. Boguslawski, P. Stremplewski, L. Kornaszewski, J. Zhang, Z. Dong, X.X. Liang, E. Gratton, A. Vogel, M. Wojtkowski, and K. Palczewski, Noninvasive two-photon optical biopsy of retinal fluorophores. Proc Natl Acad Sci U S A 117 (2020) 22532-22543.

[6] G. Palczewska, P. Stremplewski, S. Suh, N. Alexander, D. Salom, Z. Dong, D. Ruminski, E.H. Choi, A.E. Sears, T.S. Kern, M. Wojtkowski, and K. Palczewski, Two-photon imaging of the mammalian retina with ultrafast pulsing laser. JCI Insight 3 (2018).
